# A cross‐case comparison of the trauma and orthopaedic hospital experiences of adults with intellectual disabilities using interpretative phenomenological analysis

**DOI:** 10.1002/nop2.693

**Published:** 2020-12-10

**Authors:** Mary Drozd, Darren Chadwick, Rebecca Jester

**Affiliations:** ^1^ University of Wolverhampton Wolverhampton UK

**Keywords:** intellectual disability, nurses, nursing, orthopaedic, phenomenology, qualitative

## Abstract

**Aim:**

To present the cross‐case comparison component of a qualitative study exploring and describing the experiences of adults with an intellectual disability who have received trauma and orthopaedic hospital care for musculoskeletal conditions or injuries in the United Kingdom.

**Design:**

A qualitative, exploratory study was conducted using 1:1 semi‐structured interviews to describe the lived experiences of trauma and orthopaedic hospital care from the perspectives of people with intellectual disabilities and a carer of a person with profound and multiple intellectual disabilities. The data were analysed using interpretative phenomenological analysis. The Standards for Reporting Qualitative Research guidelines were applied.

**Results:**

There were common and interconnected experiences across the five participants: communication challenges; lack of person‐centred care; issues related to pain management; lack of confidence in hospital care; the valuable support and expertise of carers; and incompetence of hospital staff and isolation and loneliness.

## 1 INTRODUCTION

The level of care provided to people with intellectual disabilities (ID) in general hospitals in the United Kingdom (UK) has been an area of concern due to evidence of abuse, neglect and discrimination (Disability Rights Commission, 2006). A more recent publication by National Health Service England (NHSE) (2018), “The Learning Disabilities Mortality Review” (LeDeR Programme), reported and analysed the deaths of people with intellectual disabilities and demonstrated that many died in hospital care and on average up to 20 years younger than people without an intellectual disability.

The World Health Organization (WHO) (2020) defines intellectual disability as “*a significantly reduced ability to understand new or complex information and to learn and apply new skills (impaired intelligence). This results in a reduced ability to cope independently (impaired social functioning) and begins before adulthood, with a lasting effect on development.”*

The Global Burden of Disease Study (2016) provided evidence of the impact of musculoskeletal (MSK) conditions and highlighted the significant disability burden associated with these, which were the second highest contributor to disability. MSK conditions affect people across the life span and in all regions of the world; the most common and disabling MSK conditions were osteoarthritis, back and neck pain, fractures associated with bone fragility, injuries and systemic inflammatory conditions such as rheumatoid arthritis (WHO, 2018).

Furthermore, people with intellectual disabilities sustain more injuries, falls and accidents than the general population (Finlayson, 2011; Finlayson et al., 2010; Finlayson et al., 2014). Fractures may occur from a low impact injury if a person has osteoporosis and this places people with intellectual disabilities at an increased risk of injury following a fall (Cox et al., 2010). McCarron et al. (2011) highlight that poor dietary habits, constipation, poor mobility, low levels of exercise, low levels of vitamin D and obesity contribute to the development and high prevalence of osteoporosis in people with intellectual disabilities. A population‐based, cross‐sectional study, undertaken in Scotland, UK, concluded that the most prevalent physical health conditions affecting people with intellectual disabilities included osteoporosis, bone deformity and musculoskeletal pain (Kinnear et al., 2018). In this study comprising 1,023 participant responses, 48% of people with intellectual disabilities had MSK conditions, highlighting the high prevalence of MSK conditions and the complexity related to multimorbidity for people with intellectual disabilities.

## 2 BACKGROUND

Empirical studies investigating the experiences of people with intellectual disabilities in general hospital settings have primarily been qualitative in nature, gauging the perspectives of carers and staff more than eliciting the perspectives of people with intellectual disabilities. There were few empirical studies with participants with intellectual disabilities, yet it is their interpretation of experiences that is of paramount importance. This corpus of literature has identified a great deal of problematic practice in hospital settings. An exhaustive list of the issues identified is outside of the scope of this paper and has been reported elsewhere (reference removed for review). However, the themes identified in these studies and associated reviews include unsafe care, fear and anxiety by people with intellectual disabilities, failure of hospital staff to provide reasonable adjustments, a lack of knowledge, skills and understanding by staff, poor or negative attitudes by hospital staff towards people with intellectual disabilities, staff or system failure to adjust to the needs of people with intellectual disabilities and lack of support and understanding of the carers role with adults with intellectual disabilities in hospitals (Gibbs et al., 2008; Dinsmore, 2011; Smeltzer et al., 2012; Ali et al., 2013; Bradbury‐Jones et al., 2013; Iacono et al., 2014; Tuffrey‐Wijne, Goulding, Giatras, et al., 2014; Tuffrey‐Wijne, Goulding, Gordon, et al., 2014; Howieson, 2015; Gibbons et al., 2016; Read, et al., 2018; Read, Heslop, et al., 2018).

The systematic review conducted by Iacono et al. (2014) recommended further research focusing on specific points of encounter in hospital settings. In line with this recommendation, this qualitative empirical investigation seeks to explore the trauma and orthopaedic (T & O) hospital experiences of people with intellectual disabilities. There is currently no known published research about the lived experiences of people with intellectual disabilities in relation to T & O hospital care for MSK conditions and injuries despite them being high users of T & O services throughout their life span; therefore, this study sought to address this gap in the literature. This study focused on lived experiences of care in T & O settings, including both specialist orthopaedic hospitals and general hospitals. The research question for this study was “How do adults with an intellectual disability describe their trauma or orthopaedic hospital care?”

## 3 METHODS

### 3.1 Design

Interpretative phenomenological analysis (IPA) was an appropriate research approach for this exploratory, qualitative study which aimed to analyse the participants' experiences. Braun and Clarke (2013) assert that qualitative research puts emphasis on meanings rather than cause and effect and argue that this approach captures the complexity, disorderliness and ambiguity of the real world. As adults with intellectual disabilities have previously been excluded from health research along with evidence of inequalities and inequities in health, a transformative paradigm was appropriate (Mertens, 2009). The Standards for Reporting Qualitative Research (SRQR) guidelines have been applied to this study (O'Brien et al., 2014).

### 3.2 Method

IPA analyses and interprets (Smith et al., 2009) was adopted as this study sought to understand the meanings that the participants assigned to their T & O hospital experiences. IPA adopts an idiographic approach with the focus on the experience of each individual participant, and as such, sample numbers are typically small (Reid et al., 2005); this enables a deeper and richer analysis of each individual account to address the research aims. Moreover, the idiographic approach lent itself to the development of a rich account for each participant and was consistent with a person‐centred care conceptual framework (McCormack & McCance, 2010) which was the theoretical underpinning for the research. Person‐centredness underpinned and guided the collecting, analysing, describing and interpreting of the data throughout the study (Ravitch & Riggan, 2017) and was congruent with the theoretical underpinnings of IPA. The voices of people with intellectual disabilities were seldom present in healthcare research, despite their much poorer general health and frequent use of hospital services. Moreover, it has been argued that this exclusion from health research is due to their perceived heightened vulnerability or their inability to consent or communicate in traditional ways (Crook et al., 2015). The transformative paradigm enabled a focus on human rights and social justice within research (Mertens, 2009), which this investigation incorporates.

IPA analyses and interprets participants' experiences of a phenomenon on two levels, first at an individual level followed by second‐level analysis to identify areas of shared experiences across the participants. IPA seeks patterns in the data, and it is embedded in a phenomenological epistemology which gives experience primacy and is about understanding people's everyday experience of reality, in great detail, so as to gain an understanding of the phenomenon in question. IPA engages with a double hermeneutic cycle which relates to the participant making sense of their experience first and then the researcher making sense of the participant's experience (Smith et al., 2009). This process emphasizes the dynamic role of the researcher in interpreting the participants' experiences (Smith & Eatough, 2007). The cross‐case comparisons only are presented here as they provide another level of analysis from across the cases (Smith et al., 2009). The benefit of cross‐case comparison following analysis and interpretation of the individual accounts is that it provides an opportunity to add further credibility and confirmability of the findings as there is strength in the collective voice of the participants with an intellectual disability who are frequently unheard. Whilst not seeking to generalize by conducting the cross‐case comparison, it provided a means of looking collectively at this group of people with an intellectual disability and presenting the commonalities of their hospital experiences. Others who advocate cross‐case comparison analysis include Flowers et al. (1997) who pointed out that not every participant in their study articulated the same themes.

### 3.3 Recruitment

There were significant barriers to accessing adults with intellectual disabilities which resulted in an extended period of recruitment spanning more than a year. Sydor (2013) discussed the terms, “hard to reach” and “hidden” which apply to the participants in this study. “Hard to reach” describes a population that is difficult for researchers to access, and “hidden” refers to a population with no defined limits such that the exact size cannot be known (Sydor, 2013, p. 35). Indeed, Emerson (2011) discusses the health risks amongst the “hidden majority” of people with intellectual disabilities unknown to services highlighting the status of people in this group as often hidden both in healthcare services and heathcare research.

Participants were recruited in various ways including through the managers of local self‐advocacy groups for adults with intellectual disabilities; through national organizations that work with adults with intellectual disabilities and their healthcare professional members; and through a “Service User and Carer group” who contribute to research and teaching at a Higher Educational Institute.

### 3.4 Inclusion and exclusion criteria for participants

Participants were included if they self‐identified as having an intellectual disability, were aged 18 or over, had adequate communication abilities to participate and had experience of T & O hospital care which did not need to be within a specified time. Participants were excluded if it was deemed detrimental, for example, if the person was extremely distressed about the hospital experience or if they were unable to give informed consent. In line with the theoretical underpinnings of IPA, participants were selected purposively because this reflected a defined group for whom the research question had relevance and personal significance. It was deemed important to include a carer of an adult with profound and multiple disabilities as she served to represent her son who did not communicate through traditional verbal speech and whom we could not interview.

### 3.5 Data collection

One‐to‐one, semi‐structured interviews were conducted with five participants, and this was a useful way of gathering the lived experiences of people with intellectual disabilities (Gilbert, 2004). Four participants self‐identified as having an intellectual disability, and one participant was the mother and carer for her adult son with profound and multiple intellectual disabilities. Table 1 provides details about the participants. The audio‐recorded interviews were undertaken between May 2016–October 2016 by the first author. Four interviews took place in person at a location chosen by the participants and their advocacy manager or family carer, and one was via telephone.

**Table 1:** Details of the participants and the interviews

Names of participants	Age	Length of time since hospital experience	MSK condition or injury	Trauma or orthopaedic hospital care	Length of semi‐structured interview and adaptations	Number of pages of transcription
Kay	25	Current experiences	Current hip, knee and back pain. Total hip replacement 3 years ago.	Orthopaedic	30 min Hospital Communication Book available as a visual aid	26 pages
Ted	45	Ongoing experiences	MSK injuries following epileptic seizures. Lower limb deformity corrected by over 40 planned surgical operations during childhood	Trauma Orthopaedic	85 min Hospital Communication Book available as a visual aid Ted had a pad of paper and pen to write on during the interview. Email was used also	12 pages
Kelly	32	Current experience of preoperative assessment clinic 7 days before the interview took place	Awaiting knee surgery. Total hip replacement 6 months ago.	Orthopaedic	30 min Interview was facilitated by Nat (carer) Hospital Communication Book available as a visual aid	12 pages
Len	44	“A long time ago”	Fractured ankle bones following being hit by a car at night.	Trauma	24 min Hospital Communication Book available as a visual aid	8 pages
Sue (mother & carer)	Not known	Within the last few years 10 year ago	Fractured fingers and toes Son, Alex, had fractures to lower leg (tibia and fibula) following accident with another child who fell onto Alex.	Trauma	45 min	20 pages

Each audio recording was listened to several times prior to transcription verbatim by the first author. Table 1 illustrates the length of each interview, the number of pages of transcription and the adaptations made to facilitate and enable meaningful participation in the data collection process.

## 4 ANALYSIS

### 4.1 Data analysis and trustworthiness

The participants' idiographic accounts and subsequent themes were analysed and interpreted independently by all three authors, and consensus was reached on a case‐by‐case basis (Smith etal.,^2009^). IPA comprises both idiographic analysis of individual accounts and cross‐case comparison of themes across participants (Smith etal.,^2009^). This paper presents the latter of these analyses. See Table 1 for participant background information.

Each individual participant's superordinate (main) and subordinate (sub) themes were compared across cases resulting in the master themes (Tables 2 and 3). Here, we present the cross‐case comparison. Each master theme is described in turn and organized by the frequency with which they occurred across the narratives.

**Table 2:** Superordinate and subordinate themes across the five participants

Superordinate themes	Subordinate themes
Communication challenges	Poor explanation of hospital and MSK procedures leading to lack of comprehension
	Lack of adaptation of expressive communication and vocabulary by hospital staff leading to lack of comprehension.
	Lack of direct communication with the person with ID
	Minimal attempts to communicate with the person with ID
	People with ID not feeling listened to by hospital staff.
Lack of person‐centred care	Lack of patience and time given by hospital staff
	Feeling home discharge was premature and not well supported
	Failure to get to know the person with ID
	Inconsistency of approach and staff in MSK hospital care
	Inadequate monitoring of well‐being of patients with ID in MSK care
	Lack of sensitivity and empathy amongst hospital staff
	Inability amongst hospital staff to care holistically for people with complex support needs
Issues related to pain management	Painfulness of MSK conditions and procedures
	Lack of MSK pain assessment and monitoring in people with ID Slow or absent MSK pain management in people with ID
Lack of confidence in hospital care	Healthcare staff do not understand the support needs of people with ID
	Specialist care necessary for people with ID in hospital
	Variability in support provided by hospital staff
The valuable support and expertise of carers	Provision of ongoing essential support within the hospital setting by the carer
	Carer supporting healthcare understanding of the person with ID
	Providing essential posthospital healthcare support and monitoring for MSK treatment complications
Incompetence of hospital staff	Inability to care holistically for people with complex support needs
Isolation and loneliness	Loneliness and lack of support within the hospital setting.
	Loneliness and isolation in the home following hospital discharge
Fear of loss and dying	Carer fear that hospital care will result in the death of their family member with ID
	Carer mistrust of hospital care and decline in family member health

**Table 3:** A cross‐case comparison showing the presence of master themes for each participant and frequency across participants

Master themes across the cases	Participants				
	Kay	Ted	Kelly	Len	Sue
Communication challenges	✓	✓	✓	✓	✓
Lack of person‐centred care	✓	✓	✓	✓	✓
Issues related to pain	✓	✓	✓	✓	✘
Lack of confidence in hospital care	✓	✓	✓	✘	✓
The valuable support and expertise of carers	✓	✘	✓	✘	✓
Incompetence of hospital staff	✘	✓	✘	✘	✓
Isolation and loneliness	✘	✓	✘	✓	✘
Fear of loss/dying	✘	✘	✘	✘	✓

Several processes were used to establish trustworthiness in the study (Shenton, 2004). These included gathering and presenting a thick description of participant circumstances and characteristics, an audit trail being undertaken at each stage of the research process, and triangulation via independent coding and verification of themes by the second and third authors and reflexivity was captured in a research diary.

## 5 ETHICS

Research Ethics Committee approval for the study was granted by the university ethics committee. The principles stated in the UK Policy Framework for Health and Social Care Research (Health Research Authority (HRA) (2018) were adhered to throughout the study in line with good practice for researchers.

## 6 FINDINGS

There were five participants: Kay, Ted, Kelly, Len and Sue whose names have been changed to maintain participants' confidentiality. The themes derived from the cross‐case comparisons are interconnected. Quotations are used to illustrate and amplify the voices of the participants. The use of “…” in the participants' quotations relates to a gap in the text.

Table 2 shows the themes, and Table 3 shows the cross‐case comparisons of master themes.

### 6.1 Communication challenges

This theme was represented in all five participant accounts. Participants reported difficulties with communication whilst they were in hospital, particularly between the hospital staff and themselves.

Ted received unclear communication in hospital:

but sometimes they said things which were not true or did not make sense. (Ted)

Although the hospital staff spoke with Kay, she did not always understand the language that they used:

…I found it hard to understand… erm… long words. (Kay)

Kelly was not always communicated with directly by hospital staff, and there was a sense that this was because Kelly had Down syndrome which is synonymous with having an intellectual disability. Some hospital staff spoke to the carer rather than to Kelly which the carer perceived as being both disrespectful and an infringement on Kelly's personhood as a 32‐year‐old woman:

…and he was talking about her to her Dad. (Nat, carer for Kelly)

Nat gave another example of communication by a doctor who spoke to her rather than Kelly who could have answered the questions:

…I would say the nurses were good but the consultant, he spoke to me and not Kelly…which I really hate when Doctors do that…just stuff like how old is she…Kelly knows how old she is. (Nat, carer for Kelly)

When Len was asked about what would make his hospital stay better, he reported that staff should listen to people with disabilities and understand which suggests that this was not consistent practice in hospital:

…erm…listen to what we say… and understand. (Len)

The hospital staff tried to communicate with Alex, but these efforts were minimal overall:

…they had a go…um….and I explained to them all that… how he smiles for yes and frowns for no …yeah they did have a little bit of a go but pretty ineffective really. (Sue, Mother and carer)

All participants experienced challenges around communication whilst in T & O hospital settings.

### 6.2 Lack of person‐centred care

All participants experienced a lack of person‐centred care. Furthermore, some of the issues reported by participants raise serious concerns about quality and patient safety, which are fundamental to person‐centred care. Overlapping with the preceding master theme, this was evident in the failures to communicate well with people with intellectual disabilities in T & O care.

Kay required staff to help her to move in bed after her hip operations and she recalled an occasion where she had difficulty in conveying her wishes for the staff to stop what they were doing as more patience, time and listening were needed to provide person‐centred care to Kay:

… because um…they was trying to get me on my side and…em…and I couldn't do it…and I was trying to tell them…to stop but they wouldn't… they wouldn't stop. (Kay)

Len said that he was being discharged home before he was well enough and that he had no choice in this decision. When he was asked if there was anything that made him feel unhappy in hospital it was centred on his discharge home from hospital:

Len: going home lonely then

Interviewer: did you feel well enough when you were going home?

Len: no. (Len)

Ted said that the hospital staff did not know him as a person due to a lack of consistency in the medical staff he was seen by and lack of preparation to find out more about him from the medical notes or from the other staff:

… every appointment I have is with a new doctor…what's the point in that?…They ask me what's wrong, why don't they look at the report or ask the nurses, they must know what I come in with…

I was not always aware what was happening. (Ted)

Sue was highly dissatisfied with Alex's care in hospital because the care given was inferior for a person with an intellectual disability:

… they could treat the disabled person as a human being… rather than just a nothing. (Sue, mother and carer)

For Sue, there was indignation that the younger nurses particularly did not care holistically for Alex. They did not demonstrate the empathy, sensitivity and care Sue expected in the care of her son:

The younger generation don't know anything at all and they're harsh. (Sue, mother and carer)

Kelly recalled a time where she felt like she was treated like a child rather than an adult in hospital:

I'm 32…and I'm not a child, I'm an adult. (Kelly)

### 6.3 Issues related to pain

All the participants except Sue, the mother and carer, discussed issues related to experiencing pain whilst they were in hospital. Kelly was distressed whilst the clips were being removed from her hip wound which is known to be an uncomfortable procedure and a person with an intellectual disability would require additional time and preparation for this procedure:

I had 30 clips in my hip…I was shouting, screaming and crying…I want my Mom and Dad…the nurse did it. (Kelly)

Kay experienced uncontrolled pain in hospital, and the nurses did not take her reported pain level seriously. Kay waited a long time to receive the analgesia:

…when you say you're in pain…they say they're gonna get tablets… they don't come back for ages…and then I end up crying…because I'm in a lot of pain. (Kay)

Len experienced severe pain in hospital:

…yeah…um…very, very, very painful, yeah. (Len)

Ted also had uncontrolled pain when he was in hospital and he was not aware that he could inform the nurses about this. He was alone in a side room in the hospital, and there was no regular assessment of his pain level:

…sometimes I had very awful pain …The nurses didn't ask about the pain that often. (Ted)

### 6.4 Lack of confidence in hospital care

Four of the participants, Kay, Sue, Ted and Kelly, expressed a lack of confidence in hospital care attributable to their poor experience in hospital. Prompted by his negative experiences of hospital care, Ted went as far as to suggest there should be a special area in the hospital just for people with disabilities. He suggested that a separate area for people with ID would be better as hospital staff did not understand people with disabilities:

…it will help … to go to that special area… a proper ward and nurses who know what to do…if they put me on that ward it might have been better…someone trained to make an effort. (Ted)

There was a lack of awareness by nurses about the role of an acute liaison intellectual disability nurse (also known as “learning disability” in the UK), whose role would incorporate supporting Kelly for hospital admission and surgery. Kelly had received this support 6 months previously at the same hospital when she was preparing for the total hip replacement, but it was not made available during her second visit in preparation for knee surgery:

…Yeah, we had a LD nurse then…I know she was very good and she organised everything but we haven't had it this time. (Nat, carer for Kelly)

For Kay, she did not have confidence in the hospital care as she saw different doctors at the hospital and not the doctor who had undertaken the surgery on her hip:

… sometimes I don't see the one that does my operation… I see someone else…I'm supposed to see the one who did my operation, not, not someone else. (Kay)

Sue believed that there were insufficient resources, both physical and human, to care appropriately for Alex in hospital:

… there's usually not enough hoists and usually they're not working either. Completely hopeless… completely hopeless and regarding someone reliable to look after him while I went to the loo or anything like that… again utterly hopeless they just don't understand, you know, you say to them, you need to watch his arms cos he'll get them caught….they put the cot sides up and he gets his arms caught in them and then you have another injury to deal with. (Sue, mother and carer)

Len did not report this lack of confidence in the hospital system. He was in hospital for a relatively short period of time compared with the other participants who had many hospital experiences and longer lengths of stay than Len.

### 6.5 The valuable support and expertise of carers

Kay and Kelly discussed the paramount importance and necessity of having their carers involved in their hospital care.

There was a strong relationship between Kay and her mother, and she helped with fundamental care in hospital, including the provision of clear communication about clarifying what was happening to Kay:

**…** so sometimes they have to tell my mom and then my mom tells me what it means. (Kay)

Kelly talked about how her parents who helped her with the hip problem:

Dad … he took me there… I said, Dad my hips not moving…and after he took me to the hospital… and my Dad phoned my Mom and my Mom said stop crying, Dad will help you. (Kelly)

Ted and Len did not mention this theme which may be because both lived alone and did not have specific family or paid carers, whereas Kay and Kelly had close family carers and Sue was the mother and carer for Alex.

Finally, participants reported the importance of carers in supporting the ongoing well‐being of the person with an intellectual disability and more specifically, carer support in monitoring for complications of MSK treatments. For example, Sue noted that her son's plaster cast had cut into his leg, which he, as a person with profound and multiple intellectual disabilities, would not have been able to monitor or report it to staff:

…it was too high up on his knee and basically Alex was lifting his leg up because the cast was sawing its way through his tendon on the back of his knee. (Sue, mother & carer)

### 6.6 Incompetence of hospital staff

The experiences of both Ted and Sue highlighted a lack of competence amongst hospital staff caring for people with intellectual disabilities. Safe, competent person‐centred care was not delivered in these T & O hospital settings.

Ted reported that more support was needed in hospital from adequately trained staff that would understand and provide safe care for people with intellectual disabilities in hospital:

I had blood in my mouth… but no‐one came in…it went on for 14 hr… yeah every person had a nurse after their operation… right but I was left for 14 hr…there ought to be more support … someone that is trained properly…properly…and understands… more help from that special trained person…who comes up and explains what's happened… nurses and doctors are not trained to look after disabled people… most people in hospital don't have a disabled problem. (Ted)

Sue was the mother and carer for her son, Alex in hospital and she considered her role was fundamental, although she felt undervalued by the hospital staff. Sue stayed with her son in hospital and undertook most of his essential daily care, rarely leaving him. She was not often relieved of these caring tasks to have her basic needs met such as having food or adequate rest. However, Sue did accept the offer to have a sleep on one occasion as she believed that the nurse would replace her and stay with Alex overnight:

… I was very grateful when this nurse said I could go and have a lie down and she said she would look after Alex… She said, I'm here to look after Alex…to completely look after him. (Sue, mother & carer)

After this episode, Sue felt very disappointed as the nurse did not attend or care appropriately for Alex as Sue had expected her to and she was very reluctant to leave Alex again after that, even to eat:

… no good God no, only for the loo, absolutely hopeless…if you want something to eat, heaven help you. (Sue, mother & carer)

The hospital staff did not support or care for Sue even though she was performing a vital role in caring for Alex full‐time in hospital, instead appearing to see it as an alleviation of their own caring responsibilities.

### 6.7 Isolation and loneliness

Ted and Len both lived alone at home and discussed their feelings of isolation and loneliness both during and following their hospital admissions. They did not have a family/paid carer with them in hospital, and they were the only males in the study. Ted's account left the impression that he had been on his own in hospital and also that staff did not check on him; he was out of sight in a side room for a significant amount of time which raises serious quality of care and safety concerns:

…No‐one has ever, ever stayed with me…in a room on my own… … 14 hr I didn't see anyone. (Ted)

For Len, there was a feeling of being lonely on discharge from hospital which he did not like. Len did not want to go home and be alone as he was unable to get out of his upstairs flat for a period of time after his injury:

.…but then going home on your own, it's horrible…it's lonely. (Len)

### 6.8 Fear of loss/dying

This theme was experienced by Sue only, who was the only carer interviewed. Whilst her son Alex was in hospital, Sue was fearful that he would die if he stayed there. She believed that the hospital care was detrimental to him to the extent that she was willing to take him home prior to adequate recovery. This raises serious concerns about the quality of care and safety that is embedded in person‐centred care:

…I thought, my God, he's dying one of the carers from the college had said to me, if you need some help and so I rang him and said please just help me put him in the car so we can get out of this…so he came over and we did that and I got Alex home…He'd have died if he'd have stayed in hospital. (Sue, mother & carer)

## 7 DISCUSSION

This is the first study, as far as the authors are aware, that explores the T & O hospital experiences of people with intellectual disabilities. These experiences provide new insights into the T & O hospital care for people with intellectual disabilities who have MSK conditions and injuries. There are interconnections between the themes. However, “confidence” and “incompetence” are presented as different themes because the authors understand “incompetence” as not having the right knowledge, skills and attitudes and “confidence” as the feeling or belief that one can have faith in or rely on someone or something. Overall, the experiences reported were very poor which raises ethical, legal and professional concerns about the quality of care.

The findings herein resonate with some of the prior findings about the general hospital care of people with intellectual disabilities, highlighting challenges with communication (Bradbury‐Jones et al., 2013); poor relationships with people with intellectual disabilities in hospital (Read, Heslop, et al., 2018; Read, Williams, et al., 2018); unsafe hospital care (Gibbs et al., 2008; Tuffrey‐Wijne, Goulding, Giatras, et al., 2014; Tuffrey‐Wijne, Goulding, Gordon, et al., 2014); and the need to value the expertise and contribution of carers of people with intellectual disabilities (Tuffrey‐Wijne et al., 2016). Carers often have a vast amount of important knowledge and understanding about the person with an intellectual disability which needs to be used to enhance the quality and safety of care.

In addition, a particularly salient theme of T & O hospital care was highlighted in the experiences of the participants: the management of pain. Pain is well known to be a prevalent symptom in people with MSK conditions and injuries (Mackintosh‐Franklin, 2014). A decade ago, Webber et al. (2010) found that hospital staff did not manage pain in people with intellectual disabilities effectively. However, in their study, the concern was expressed by carers of older people with intellectual disabilities in Australia. Tuffrey‐Wijne et al. (2016) add that people with intellectual disabilities may have challenging behaviours which might mask symptoms of pain; therefore, the importance of recognizing “unconventional” ways of expressing pain is essential. Moreover, Cooper et al. (2014) contend that if nurses are unaware of specific assessment tools for use with people with intellectual disabilities then pain may be missed, and distress increased. There are tools to assist staff in assessing pain when people with intellectual disabilities cannot communicate verbally, such as the Disability Distress Assessment Tool (DISDAT) (Regnard et al., 2007). This tool, used in conjunction with carers' knowledge and appropriate training, can be used to minimize unnecessary pain.

### 7.1 Quality of care

The reported experiences from the participants demonstrate a lack in the quality of care provided alongside serious safety issues in T & O hospital settings. Quality and safety are key components of person‐centred care (Santana et al., 2018).

The current political agenda is directed towards inclusion rather than segregation of people with intellectual disabilities in general hospital care, but this appears to be failing to meet the specific needs of this disadvantaged group of people. Given the ratification of the United Nations Convention on the Rights of Persons with Disabilities (United Nations, 2006), it would appear from the accounts in this study that hospital care is failing a specific group of individuals. Moreover, the recent response to the COVID‐19 pandemic highlights that there is still a long way to go to value the lives of people with an intellectual disability as the National Institute for Health and Care Excellence (NICE) (2020) rapidly updated the rapid COVID‐19 critical care guidelines to clarify and emphasize the need to consider additional patient factors with patients with intellectual disabilities. Prior to the amendment of the NICE guideline, people with intellectual disabilities were not eligible for critical care during the pandemic.

### 7.2 Strengths and limitations

Inclusion of adults with intellectual disabilities as participants is a particular strength of this investigation. However, not all people with intellectual disabilities attend self‐advocacy groups or will be in receipt of intellectual disability services; therefore, the participants recruited did not include the “hidden majority” of people with intellectual disabilities (Emerson, 2011). Nevertheless, the aims of this study were to illuminate the experiences of the participants rather than to represent all people with intellectual disabilities who are not a homogenous group (Newell & Burnard, 2010). The accounts provide valuable insights, and further research is needed to elaborate on the findings here.

This qualitative investigation relied on people's memories to describe their past experiences with interviews conducted after the hospital stay. Memory is both changeable and constructed (Barusch, 2011; Blakey et al., 2019; Young Rojahn, 2013), and the accuracy of memory of people with intellectual disabilities has often been questioned (Morales et al., 2017). However, Cohen et al. (2010) contend that what people remember is significant to them and it has been acknowledged that assumptions should not be made that the memory of people with intellectual disabilities is poor; Morales et al. (2017) demonstrated that emotional memory of hospital experiences in people with intellectual disabilities are stable over time. Blakey et al. (2019) contend that it is acceptable in qualitative research to value experiences and feelings over any alleged accuracy of memory and this was the stance adopted in the present study. Moreover, interviewing people with intellectual disabilities after their hospital experience rather than during the hospitalization was considered less distressing for participants. Although the study sample maybe considered small it is consistent with IPA approaches which focus on in‐depth experiences of individual and cross‐case analysis and interpretation (Smith et al., 2009).

### 7.3 Relevance to clinical practice

The estimated number of people who have an intellectual disability globally is rising and as high volume users of trauma and orthopaedic services this is a significant issue for healthcare providers; this research is providing new and valuable insights into this area of practice from the perspective of adults with intellectual disabilities and a mother/carer of an adult with profound and multiple intellectual disabilities.

This study reveals that adults with intellectual disabilities did not have their pain managed effectively; they did not understand what was happening to them in T & O hospital settings, and they were neglected at times. Furthermore, people with intellectual disabilities can participate effectively in research studies and make valuable contributions to the evidence base.

## 8 CONCLUSION

This is the first known study that has investigated T & O hospital care from the perspectives of adults with an intellectual disability and a carer of an adult with profound and multiple intellectual disabilities. The participants' descriptions of their experiences raise concerns about the quality of care and patient safety. There was a distinct lack of person‐centred care, fundamental pain assessment and management were poor resulting in unnecessary suffering, and there were communication challenges which caused anxiety and episodes of unsafe care such as being left alone for long periods in a side room without being monitored postsurgery which amounts to neglect. The family and paid carers were highly valued by the people with an intellectual disability in the T & O hospital setting.

This new knowledge provides valuable insights and enriches the evidence base about the T & O hospital experiences from the perspectives of people with intellectual disabilities and a family carer. It is recommended that there is urgent action by healthcare organizations that are responsible for training and educating hospital staff to provide person‐centred care for people with intellectual disabilities. In particular, effective communication to conduct holistic assessments along with valuing people with intellectual disabilities and their carers is of paramount importance. Further research will be needed with people with intellectual disabilities, who are experts by lived experience and well placed to co‐deliver and evaluate the impact of this education and training.

## CONFLICT OF INTEREST

There is no conflict of interest to declare.

## Data Availability

The data that support the findings of this study are available in WIRE: (Wolverhampton Intellectual Repository and E‐Theses). WIRE is the University of Wolverhampton's open‐access collection of research outputs produced within its research centres and institutes. http://hdl.handle.net/2436/623751.
